# Long-Term Stability of Pickering Nanoemulsions Prepared Using Diblock Copolymer Nanoparticles: Effect of Nanoparticle Core Crosslinking, Oil Type, and the Role Played by Excess Copolymers

**DOI:** 10.1021/acs.langmuir.2c00821

**Published:** 2022-06-23

**Authors:** Saul J. Hunter, Steven P. Armes

**Affiliations:** Department of Chemistry, Dainton Building, University of Sheffield, Brook Hill, Sheffield, South Yorkshire S3 7HF, U.K.

## Abstract

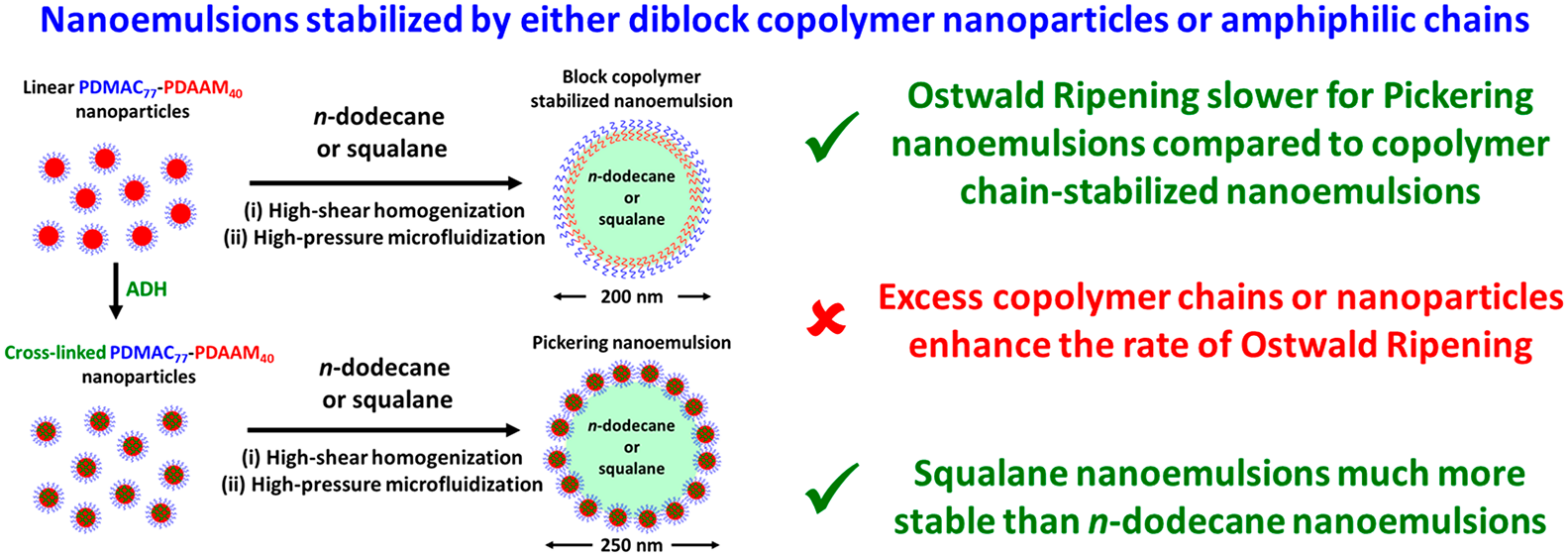

A poly(*N*,*N*′-dimethylacrylamide) (PDMAC) precursor is chain-extended via reversible addition–fragmentation chain transfer (RAFT) aqueous dispersion polymerization of diacetone acrylamide (PDAAM) to produce PDMAC_77_-PDAAM_40_ spherical nanoparticles. Post-polymerization core-crosslinking of such nanoparticles was performed at 20 °C, and the resulting covalently stabilized nanoparticles survive exposure to methanol. The linear and core-crosslinked nanoparticles were subjected to high-shear homogenization in turn in the presence of *n*-dodecane to form macroemulsions. Subsequent processing of these macroemulsions via high-pressure microfluidization produced nanoemulsions. When using the core crosslinked nanoparticles, the droplet diameter was strongly dependent on the copolymer concentration. This indicates that such nanoparticles remain intact under the processing conditions, leading to formation of genuine Pickering nanoemulsions with a *z*-average diameter of 244 ± 60 nm. In contrast, the linear nanoparticles undergo disassembly to afford molecularly dissolved diblock copolymer chains, which stabilize oil droplets of 170 ± 59 nm diameter. The long-term stability of these two types of *n*-dodecane-in-water nanoemulsions with respect to Ostwald ripening was examined using analytical centrifugation. When prepared at the same copolymer concentration, Pickering nanoemulsions stabilized by core-crosslinked nanoparticles proved to be significantly more stable than the nanoemulsion stabilized by the amphiphilic PDMAC_77_-PDAAM_40_ chains. Moreover, higher copolymer concentrations led to a significantly faster rate of droplet growth. This is attributed to excess copolymer facilitating the diffusion of *n*-dodecane through the aqueous phase. Finally, analytical centrifugation is used to assess the long-term stability of the analogous squalane-in-water nanoemulsions. These systems are much more stable than the corresponding *n*-dodecane-in-water nanoemulsions, regardless of whether the copolymer is adsorbed as sterically stabilized nanoparticles or surface-active chains.

## Introduction

Particle-stabilized emulsions were first reported by Ramsden in 1903.^1^ Subsequent more widely recognized studies by Pickering led to them being described as “Pickering emulsions” in the literature.^2^ In principle, such emulsions offer reduced foaming problems, greater stability, and more reproducibility compared to surfactant-stabilized emulsions.^3^ With the exception of a few under-appreciated industrial applications, the field of Pickering emulsions remained largely neglected for almost a century.^4^ However, pioneering studies by Binks and others have reignited academic interest in this topic.^3,5^ Pickering emulsions can be prepared using various types of particles.^6−9^ Indeed, it is now widely recognized that particle surface chemistry is much more important than the bulk composition. More specifically, the particle contact angle (or particle wettability) dictates whether a Pickering emulsion will be of the oil-in-water (o/w) or water-in-oil (w/o) type.^3,10−12^ Together with the particle size, this parameter also influences the emulsion stability.^3,6,10,13,14^

Nanoemulsions comprise relatively fine oil or water droplets.^15−17^ The upper limit droplet diameter for a genuine nanoemulsion is a matter of some debate in the literature, with values ranging from 200 to 500 nm.^15,18^ However, it is generally agreed that, like conventional emulsions, nanoemulsions are only kinetically stable.^17,19^ Moreover, their relatively small size leads to a high Laplace pressure, which means that they are rather susceptible to droplet growth via Ostwald ripening.^20,21^ In principle, this can be suppressed for oil-in-water nanoemulsions by selecting an oil with a relatively low aqueous solubility. In practice, droplet growth over time periods of days/weeks is observed even for *n*-alkanes.^9,20,22^ Typically, nanoemulsions are prepared using surfactants,^23,24^ amphiphilic diblock copolymers,^25^ or inorganic nanoparticles such as silica sols.^9,26^ In the latter case, Pickering nanoemulsions can be obtained.

There has been considerable interest in polymerization-induced self-assembly (PISA) over the last two decades.^27^ Essentially, PISA involves the growth of an insoluble polymer chain from one end of a soluble polymer chain to produce an amphiphilic diblock copolymer in a selective solvent. Growth of the insoluble block initially leads to micellar self-assembly and eventually produces sterically stabilized diblock copolymer nanoparticles. The most common copolymer morphology is spheres, and the mean particle diameter can be adjusted by systematic variation of the relative volume fraction of each block.^11,28^ Notably, PISA enables the preparation of diblock copolymer nanoparticles that are sufficiently small (i.e., 20–30 nm diameter) to allow the stabilization of Pickering nanoemulsions.^22,29−31^ If such nanoparticles are prepared in aqueous media, they are inherently hydrophilic (particle contact angle < 90°) and therefore favor the formation of oil-in-water Pickering nanoemulsions.^22,29,30^ Normally, such nanoparticle syntheses involve reversible addition–fragmentation chain transfer (RAFT) aqueous emulsion polymerization since the water-immiscible monomer (e.g., benzyl methacrylate or 2,2,2-trifluoroethyl methacrylate) ensures that the core-forming block is highly hydrophobic.^11,28,29^ This is an important consideration for the survival of the nanoparticles under the high-pressure microfluidization conditions required to generate Pickering nanoemulsions from initial coarse Pickering macroemulsions. Indeed, nanoparticles prepared via RAFT aqueous dispersion polymerization of a water-miscible monomer (e.g., 2-hydroxypropyl methacrylate) typically do not survive such high-energy processing conditions because the corresponding core-forming block is only weakly hydrophobic.^32,33^ Instead, disassembly produces amphiphilic copolymer chains, which then act as an emulsifier.^11,34^ However, such nanoemulsions are not genuine Pickering nanoemulsions.

**Figure 1:**
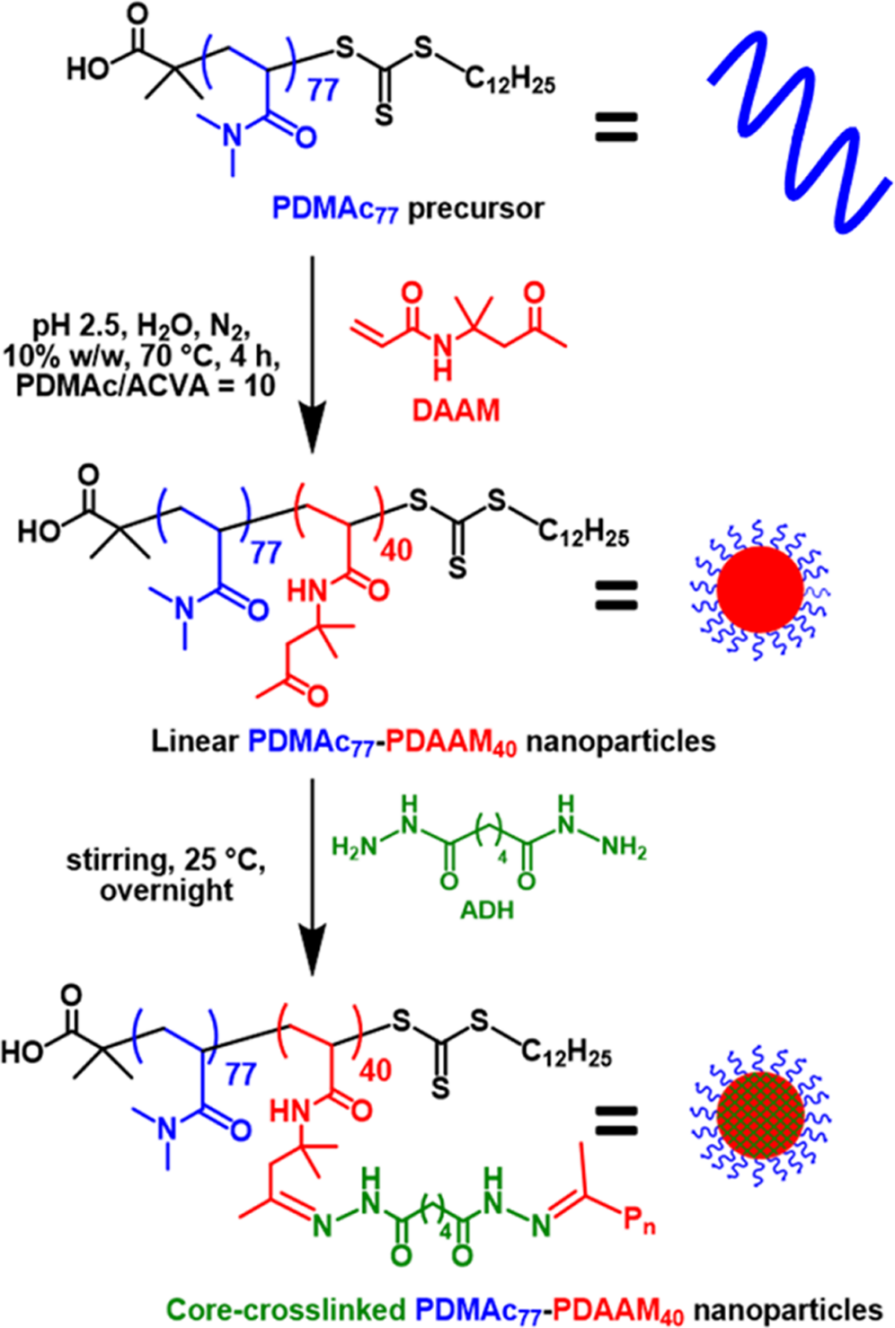
Synthesis of linear PDMAC_77_-PDAAM_40_ diblock copolymer nanoparticles via RAFT aqueous dispersion polymerization of DAAM. Subsequent covalent stabilization of such nanoparticles was achieved using ADH to form hydrazone linkages between the PDAAM chains within the nanoparticle cores.

Herein, we use a well-documented RAFT aqueous dispersion polymerization formulation^35^ to prepare sterically stabilized diblock copolymer nanoparticles in which the hydrophilic block is poly(*N*,*N*′-dimethylacrylamide) (PDMAC) and the hydrophobic block is poly(diacetone acrylamide) (PDAAM) (Figure 1). As expected, the linear nanoparticles do not survive high-pressure microfluidization but nanoemulsion droplets are nevertheless stabilized by the amphiphilic PDMAC_77_-PDAAM_40_ chains. In contrast, crosslinking the PDAAM chains within the nanoparticle cores using adipic acid dihydrazide (ADH) enables the production of genuine Pickering nanoemulsions. The long-term stability exhibited by these two types of nanoemulsions is compared for two oils (*n*-dodecane and squalane) using analytical centrifugation. Moreover, the deleterious effect of excess copolymer on the rate of droplet growth is demonstrated.

## Results and Discussion

**Figure 2:**
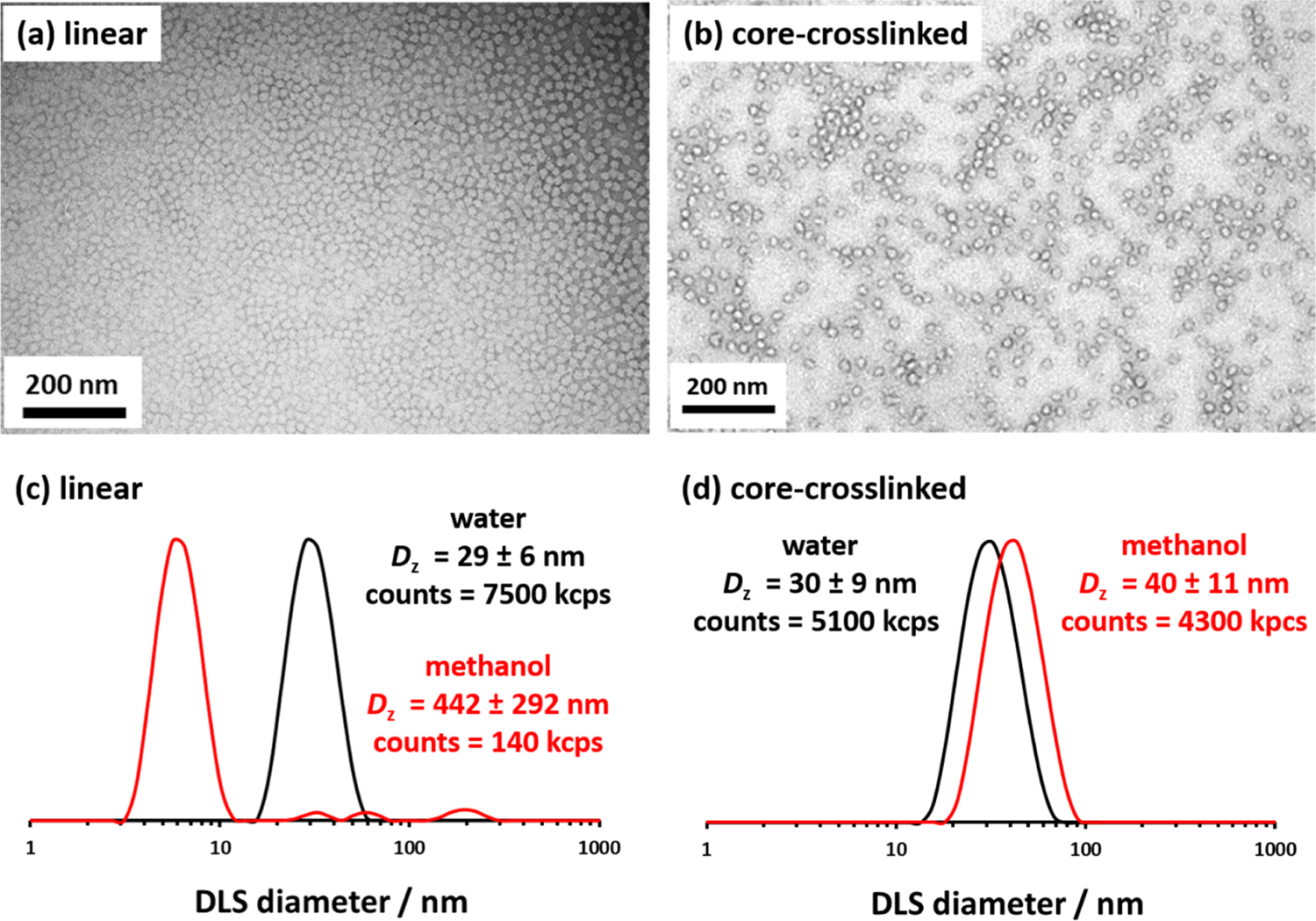
TEM images and corresponding DLS data were recorded for linear and core crosslinked PDMAC_77_-PDAAM_40_ nanoparticles in methanol and water, respectively.

A PDMAC_77_ precursor was chain-extended via RAFT aqueous dispersion polymerization of DAAM to produce diblock copolymer nanoparticles when targeting 10% w/w solids.^35^ Importantly, the solution pH was adjusted to pH 2.5 to prevent ionization of the terminal carboxylic acid groups on the PDMAC chains during polymerization. Byard and co-workers conducted kinetic studies on such polymerizations and found that essentially full DAAM conversion was obtained after 100 min at 70 °C.^35^ Moreover, well-defined spherical nanoparticles were invariably formed if the PDMAC stabilizer block was sufficiently long (mean DP ≥ 68). Based on our experience, relatively small nanoparticles (<30 nm diameter) are required to prepare Pickering nanoemulsions.^29^ Accordingly, a relatively long PDMAC_77_ precursor was chain-extended with a short PDAAM_40_ block to form sufficiently small spherical nanoparticles. When targeting 10% w/w solids, ^1^H NMR spectroscopy studies confirmed that at least 99% DAAM conversion was obtained within 100 min at 70 °C (Figure S1). Gel permeation chromatography (GPC) analysis indicated that this RAFT polymerization was relatively well-controlled (*M*_w_/*M*_n_ = 1.30) (Figure S2). A transmission electron microscopy (TEM) image indicating a well-defined spherical morphology for the final PDMAC_77_-PDAAM_40_ nanoparticles is shown in Figure 2. Dynamic light scattering (DLS) was used to determine a *z*-average diameter of 29 ± 6 nm, which is consistent with the number-average diameter of 14 ± 3 nm estimated by TEM via digital image analysis of at least 100 nanoparticles. In addition to the effect of polydispersity, the latter technique is only sensitive to the nanoparticle cores, whereas the former technique is sensitive to the overall hydrodynamic diameter of these sterically stabilized nanoparticles.

Such diblock copolymer spheres were covalently stabilized using ADH, as reported by Byard and co-workers.^35^ This bifunctional reagent forms hydrazone crosslinks between the PDAAM chains. Byard and co-workers reported that utilizing an ADH/DAAM molar ratio of 0.10 produced sufficient crosslinking to prevent nanoparticle disassembly on dilution with methanol (a good solvent for both blocks). Hence, the same molar ratio was used for the PDMAC_77_-PDAAM_40_ nanoparticles described herein, with crosslinking conducted for 16 h at 25 °C. TEM images recorded for the linear and core-crosslinked PDMAC_77_-PDAAM_40_ spheres are shown in Figure 2. As expected, DLS studies confirmed that the core-crosslinked nanoparticles did not undergo disassembly when diluted with methanol. Instead, there was a modest increase in size, with a slight reduction in the derived count rate (from 5100 kpcs in water to 4300 kcps in methanol), as shown in Figure 2d. These observations are consistent with the formation of methanol-swollen core-crosslinked nanoparticles. In contrast, there is a significant reduction in the derived count rate when diluting the linear nanoparticles with methanol (from 7500 kpcs in water to 140 kcps in methanol), which indicates the formation of molecularly dissolved copolymer chains in this case (Figure 2c).^36^ Indeed, the number-average diameter decreases from 21 to 5 nm under such conditions, which suggests that nanoparticle disassembly has occurred.

Linear and crosslinked PDMAC_77_-PDAAM_40_ nanoparticles were employed over a range of copolymer concentrations to prepare a series of macroemulsions via high-shear homogenization. More specifically, these aqueous dispersions were mixed with *n*-dodecane (50% by volume) and then homogenized at 13,500 rpm for 2 min at 20 °C. Previously, Thompson et al. found that genuine Pickering emulsions are typically not obtained when employing diblock copolymer nanoparticles comprising weakly hydrophobic cores.^32,37^ This is because *in situ* nanoparticle dissociation occurs during emulsification, resulting in oil droplets stabilized by amphiphilic copolymer chains. In such cases, laser diffraction studies confirmed that the droplet size is essentially independent of the copolymer concentration. In contrast, the droplet diameter of a genuine Pickering emulsion is strongly concentration-dependent, which is indeed observed when using the analogous covalently stabilized nanoparticles. Figure S3 shows laser diffraction data obtained for the resulting *n*-dodecane-in-water macroemulsions. Systematically reducing the copolymer concentration leads to a gradual increase in the volume-average droplet diameter for emulsions prepared using the core-crosslinked nanoparticles. This well-known behavior indicates that the nanoparticles survive high-shear emulsification to form genuine Pickering emulsions.^32,34^ In the case of the linear spherical nanoparticles, smaller droplets of 5–10 μm diameter were observed. Furthermore, a significantly weaker concentration dependence was observed for this parameter compared to that observed for the crosslinked nanoparticles (Figure S3). Such behavior is characteristic of soluble copolymer emulsifiers, suggesting that the linear nanoparticles undergo disassembly to generate amphiphilic diblock copolymer chains during homogenization.^38^

**Figure 3:**
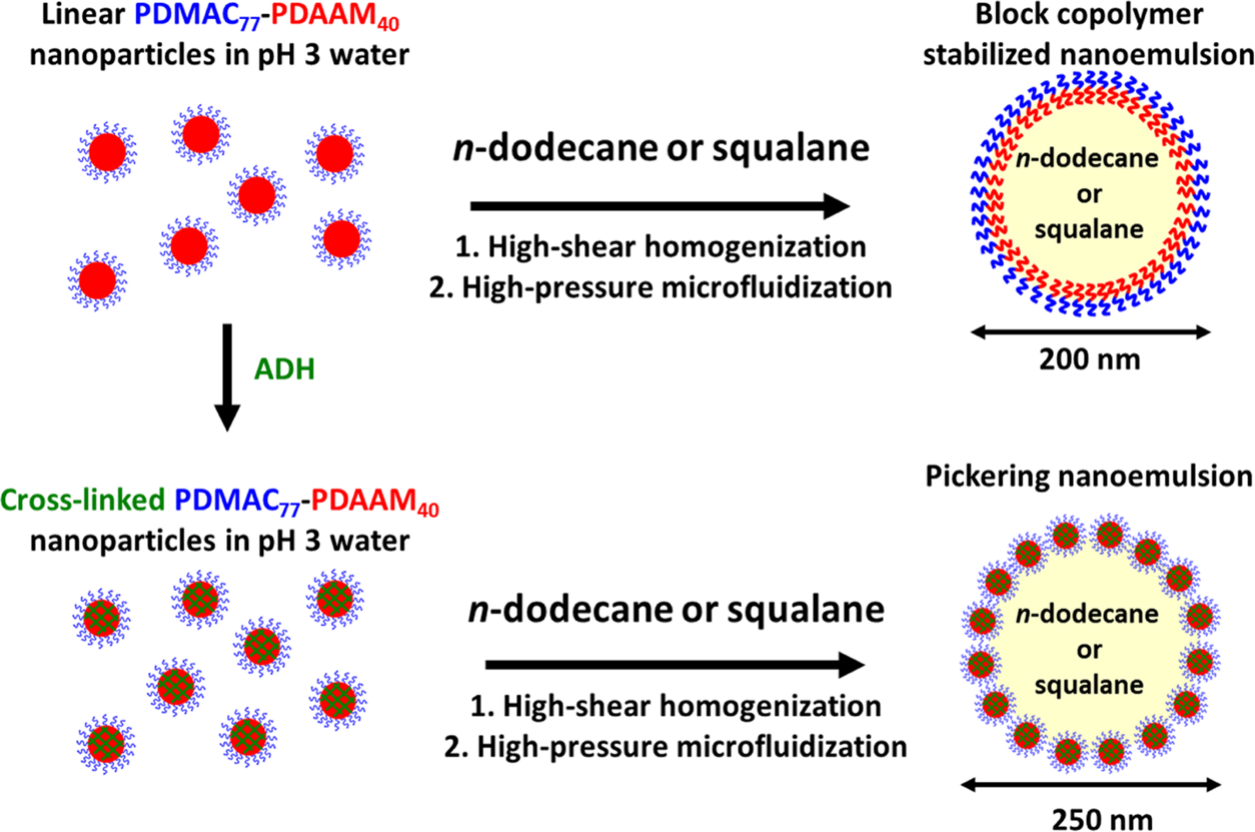
Schematic preparation of nanoemulsions using either linear or core-crosslinked PDMAC_77_-PDAAM_40_ nanoparticles. First, an aqueous dispersion of either linear or core-crosslinked nanoparticles at pH 3 are homogenized with *n*-dodecane to form an *n*-dodecane-in-water precursor macroemulsion of around ∼10 μm diameter using conventional high-shear homogenization at 13,500 rpm for 2 min at 20 °C. Subsequently, this macroemulsion is then subjected to eight passes through an LV1 microfluidizer at 30,000 psi to obtain nanoemulsions of approximately 200–250 nm diameter. When using core-crosslinked nanoparticles, genuine Pickering nanoemulsions are produced. In contrast, using linear nanoparticles leads to nanoemulsions stabilized by individual amphiphilic copolymer chains owing to disassembly during high-shear microfluidization.

**Figure 4:**
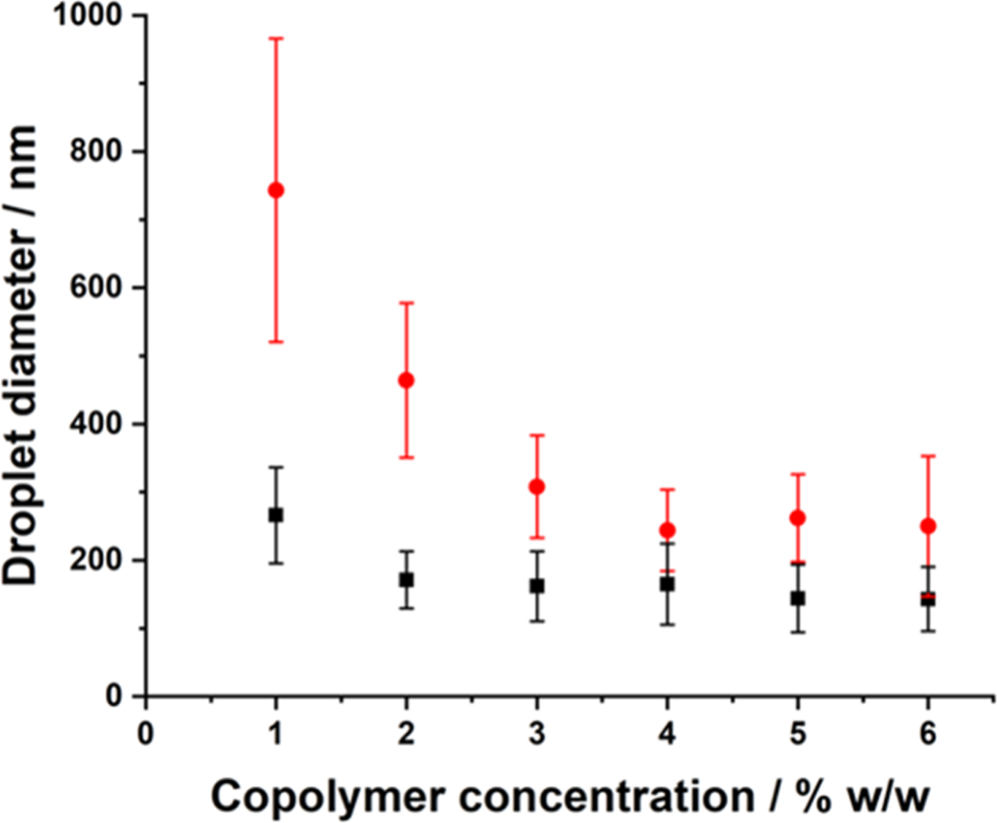
Copolymer concentration dependence of the DLS droplet diameter for two series of *n*-dodecane-in-water nanoemulsions prepared using either core-crosslinked (red circles) or linear (black squares) PDMAC_77_-PDAAM_40_ spherical nanoparticles after eight passes at an applied pressure of either (a) 10,000 psi or (b) 30,000 psi.

Subsequently, either linear or core-crosslinked nanoparticles were used to prepare a range of Pickering macroemulsions using higher copolymer concentrations (1–6% w/w) at a constant oil volume fraction of 0.20. The large excess of nanoparticles within the aqueous phase of this macroemulsion is essential for the second step: their adsorption stabilizes the (much greater) additional interfacial area created during microfluidization to generate the final Pickering nanoemulsion.^9,29^ Such precursor macroemulsions were then subjected to eight passes through a microfluidizer at 30,000 psi (Figure 3). We have recently reported that minimal surface charge arising from the ionization of terminal carboxylic acid groups can inhibit the adsorption of sterically stabilized nanoparticles and hence compromise the nanoemulsion stability.^30^ To prevent this problem, a 10% w/w aqueous dispersion of PDMAC_77_-PDAAM_40_ nanoparticles was diluted to the desired copolymer concentration using a mildly acidic aqueous solution (pH 3) prior to homogenization. This protocol ensured that the terminal carboxylic acid groups (p*K*_a_ ∼ 5) on each PDMAC steric stabilizer chain remained protonated during dilution.^39^ In principle, smaller droplets should be formed when using higher nanoparticle concentrations since more nanoparticles are available to adsorb onto the new (much smaller) oil droplets created during microfluidization.^8,14,34^ However, if the nanoparticles dissociate to form molecularly dissolved copolymer chains during emulsification, then the droplet diameter typically remains more or less constant when increasing the nanoparticle concentration.^14^ This is because only a relatively small amount of copolymer chains is required to produce the minimum droplet diameter. Indeed, almost no change in the *z*-average droplet diameter occurs when varying the copolymer concentration in the case of the linear PDMAC_77_-PDAAM_40_ nanoparticles (Figure 4). This provides indirect evidence that the high-pressure microfluidization conditions required to prepare nanoemulsions can lead to *in situ* disassembly. Such instability is consistent with our prior studies.^32,33^

**Figure 5:**
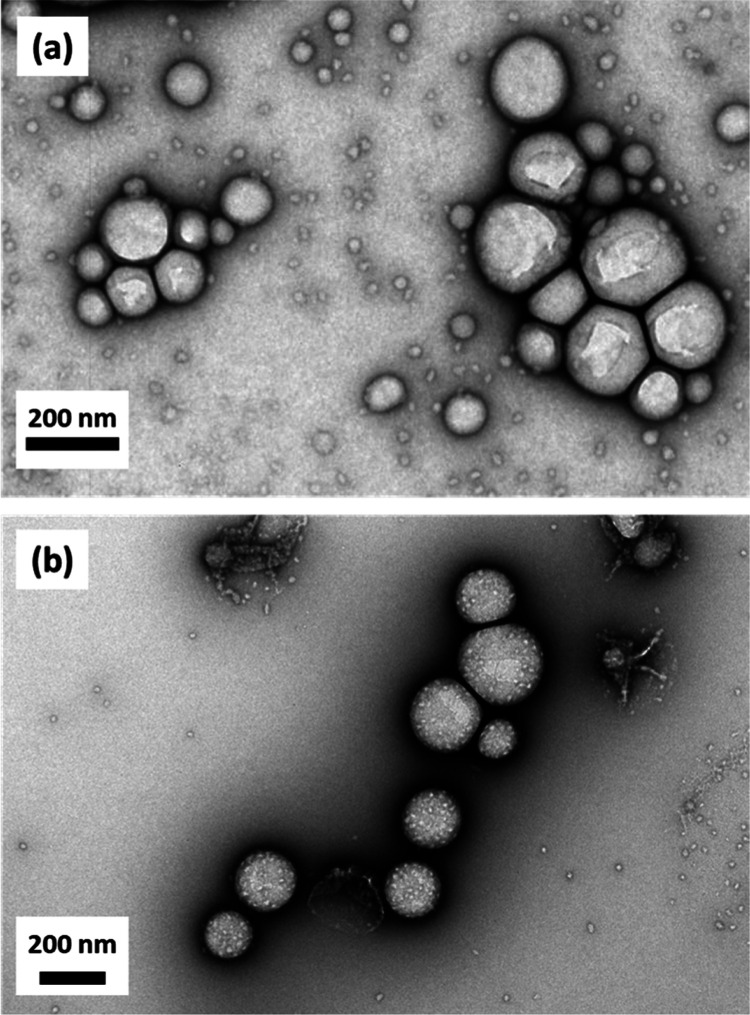
TEM images obtained for dried *n*-dodecane-in-water nanoemulsions prepared using either (a) linear or (b) core-crosslinked PDMAC_77_-PDAAM_40_ spherical nanoparticles.

To establish whether the nanoparticles survived the energy-intensive microfluidization conditions intact, dried nanoemulsion droplets (prepared using either linear or core-crosslinked nanoparticles at 30,000 psi) were imaged by TEM. Under ultrahigh vacuum conditions, evaporation of both the *n*-dodecane droplets and the aqueous continuous phase occurs, leaving only the non-volatile copolymer component on the TEM grid. When using the linear PDMAC_77_-PDAAM_40_ nanoparticles, TEM studies reveal the presence of polydisperse spheres whose size corresponds approximately to the DLS diameter observed for the original nanoemulsion (Figure 5a). Clearly, there is no evidence for the original nanoparticles within these spheres, which exhibit a smooth, featureless structure. This suggests that the linear nanoparticles do indeed undergo disassembly during microfluidization. In contrast, the original core-crosslinked nanoparticles remain clearly visible within shell-like superstructures, indicating that this was a genuine Pickering nanoemulsion prior to its exposure to the UHV conditions needed for TEM studies (Figure 5b).

Pickering nanoemulsions prepared using sterically stabilized nanoparticles are relatively unstable when *n*-dodecane is utilized as the droplet phase.^22,29^ In the present study, we seek to compare the instability of such nanoemulsions with those prepared using the corresponding diblock copolymer chains. Accordingly, a Pickering macroemulsion was prepared by homogenizing a 5% w/w dispersion of core crosslinked PDMAC_77_-PDAAM_40_ nanoparticles with 20% *n*-dodecane (by volume). The same chain-stabilized emulsion was also prepared using linear PDMAC_77_-PDAAM_40_ nanoparticles. These macroemulsions were then passed through the microfluidizer eight times at 30,000 psi. This pressure was selected to ensure that the linear nanoparticles disintegrated during microfluidization to form amphiphilic diblock copolymer chains. Thus, this strategy produces two nanoemulsions prepared using emulsifiers with almost identical chemical compositions at the same oil volume fraction and copolymer concentration. The key difference is the physical nature of the emulsifier. Thompson and co-workers also showed that nanoemulsions stabilized by diblock copolymer chains could be prepared from PGMA_48_-PTFEMA_50_ nanoparticles if the applied pressure was sufficiently high.^29^ However, the long-term stability of such nanoemulsions was not explored.

For nanoemulsions, it is well known that Ostwald ripening is the main destabilization mechanism.^9,22,40,41^ Lifshitz and Slyozov^42^ and Wagner^43^ independently developed a quantitative LSW theory for Ostwald ripening. This assumes that the dispersed phase comprises spherical droplets whose interseparation distance is significantly greater than the mean droplet diameter. Moreover, mass transport is considered to be limited by molecular diffusion through the continuous phase. If these assumptions are valid, then the rate of Ostwald ripening, ω, is given by eq 1
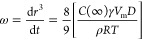
1where *C*(∞) is the solubility of the dispersed phase within the continuous phase, *D* is the diffusion coefficient for the molecularly dissolved species in the continuous phase, *V*_m_ is the molar volume of the droplet phase, ρ is the density of the droplets, and *r* is the droplet radius. If the predominant instability mechanism involves Ostwald ripening, then eq 1 predicts that a plot of *r*^3^ vs time should be linear. Accordingly, analytical centrifugation was employed to monitor the change in the volume-average droplet radius (*r*) over time for nanoemulsions stabilized by either nanoparticles or chains.

**Figure 6:**
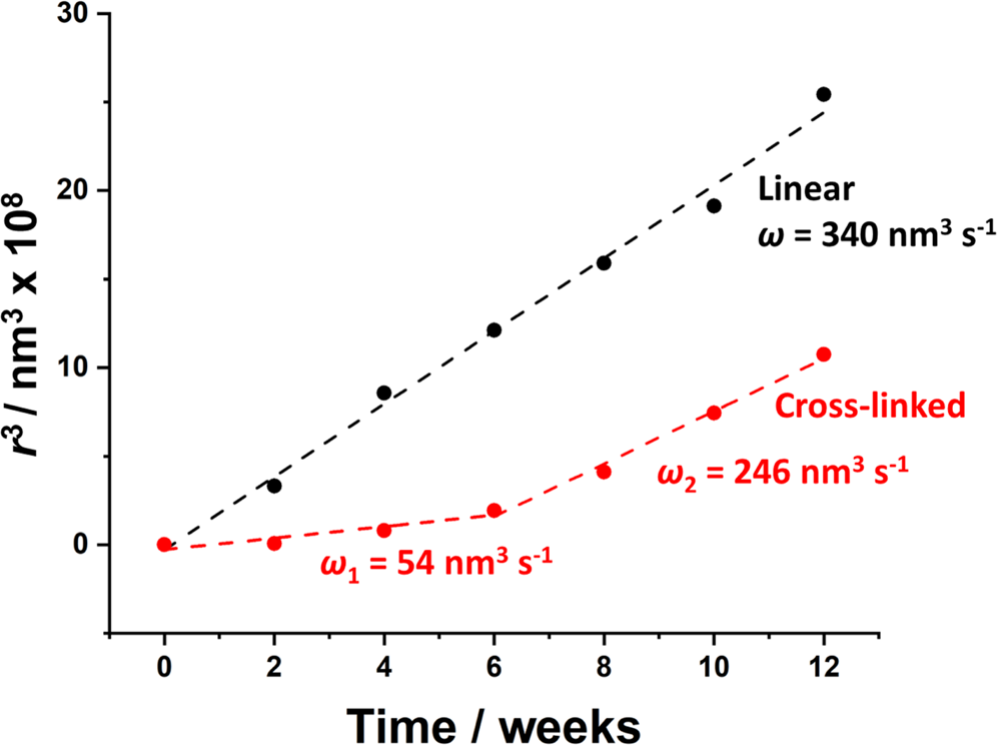
Time dependence of the cube of the mean droplet radius (*r*^3^) at 20 °C when aging *n*-dodecane-in-water nanoemulsions prepared with either core-crosslinked PDMAC_77_-PDAAM_40_ nanoparticles (red circles) or linear diblock copolymer chains (black circles). A linear relationship is observed for the droplet growth of nanoemulsions prepared using the diblock copolymer chains, suggesting an Ostwald ripening destabilization mechanism. The Pickering nanoemulsions prepared using the core-crosslinked nanoparticles also undergo Ostwald ripening but a pronounced change in gradient is observed after approximately 6 weeks.

For the nanoemulsions prepared using the non-crosslinked nanoparticles (which undergo *in situ* disintegration to afford copolymer chains), a linear *r*^3^ vs time plot was observed over a 12-week period (Figure 6). This suggests that the growth of such droplets occurs via Ostwald ripening. However, Pickering nanoemulsions prepared using the core-crosslinked nanoparticles exhibit different behavior. Initially, the plot of *r*^3^ against time is linear, albeit with a significantly lower gradient (54 vs 340 nm^3^ s^–1^). After approximately 6 weeks, the nanoemulsion becomes significantly less stable, with droplet growth following a significantly steeper gradient of 246 nm^3^ s^–1^. This suggests that additional factors most likely influence the rate of droplet growth in this case. Nevertheless, the Pickering nanoemulsion is less unstable than the nanoemulsion prepared using the amphiphilic diblock copolymer chains. Presumably, the nanoparticles adsorbed at the *n*-dodecane–water interface act as a physical barrier and hence hinder oil diffusion into the aqueous phase, thus reducing the rate of Ostwald ripening. We recently reported similar stability differences for two Pickering nanoemulsions prepared using either charged or neutral nanoparticles.^30^ Since the charged nanoparticles were much more loosely packed around the *n*-dodecane droplets than the neutral nanoparticles, the former nanoemulsion exhibited poorer stability.

For the copolymer chain-stabilized nanoemulsions reported herein, the minimum droplet diameter is more or less independent of copolymer concentration at 30,000 psi (Figure 4). Thus, the aqueous continuous phase is highly likely to contain excess non-adsorbed diblock copolymer chains. It is well documented that excess surfactant is detrimental to long-term nanoemulsion stability.^20,44,45^ This was explained in terms of the micelle-assisted transport of oil between droplets and a lower Gibbs elasticity.^45^ Given the amphiphilic nature of the PDMAC_77_-PDAAM_40_ copolymer chains, this explanation may also account for the comparatively poor stability observed in the present study.

**Figure 7:**
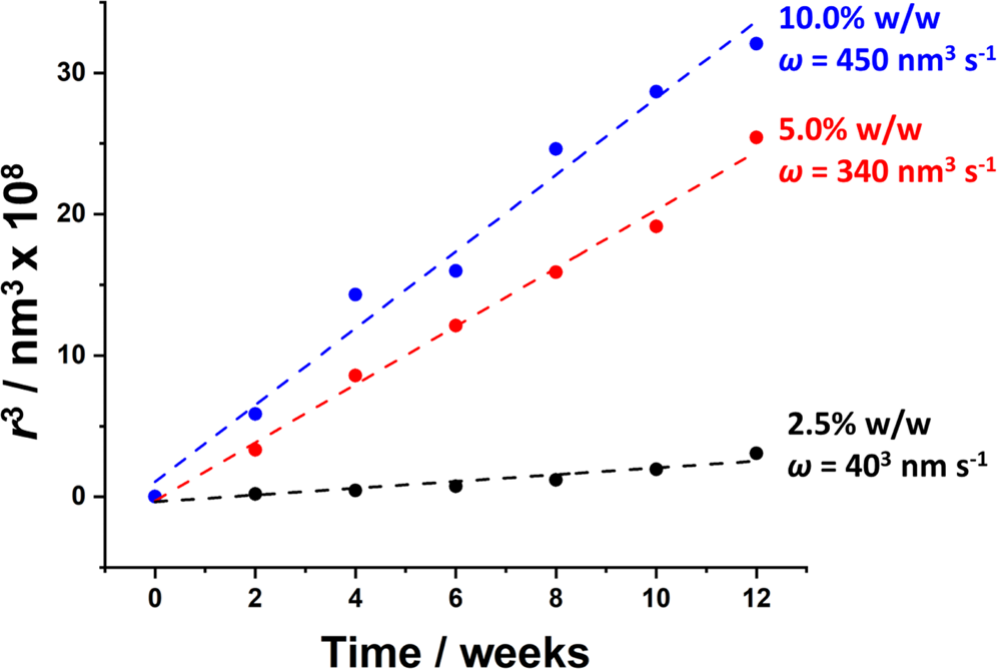
Time dependence of the cube of the mean droplet volume-average radius (*r*^3^) of *n*-dodecane-in-water nanoemulsions aged at 20 °C. These nanoemulsions were prepared using linear PDMAC_77_-PDAAM_40_ chains (red circles) at copolymer concentrations of 2.5% w/w (black), 5.0% w/w (red), and 10% w/w (blue).

To explore the effect of copolymer concentration on long-term stability, a new series of nanoemulsions were prepared using copolymer concentrations ranging from 2.5 to 10% w/w. Droplet growth was monitored over 12 weeks using analytical centrifugation (Figure 7). As expected, *r*^3^ increased linearly over time for all three copolymer concentrations, indicating that such nanoemulsions coarsen predominantly via an Ostwald ripening mechanism. From these linear gradients, the Ostwald ripening rates were calculated to be 40, 340, and 450 nm^3^ s^–1^ when using copolymer concentrations of 2.5, 5.0, and 10.0% w/w, respectively. Thus, using a higher copolymer concentration (and hence having a larger excess of non-adsorbed amphiphilic copolymer chains in the aqueous phase) produces faster Ostwald ripening.

**Figure 8:**
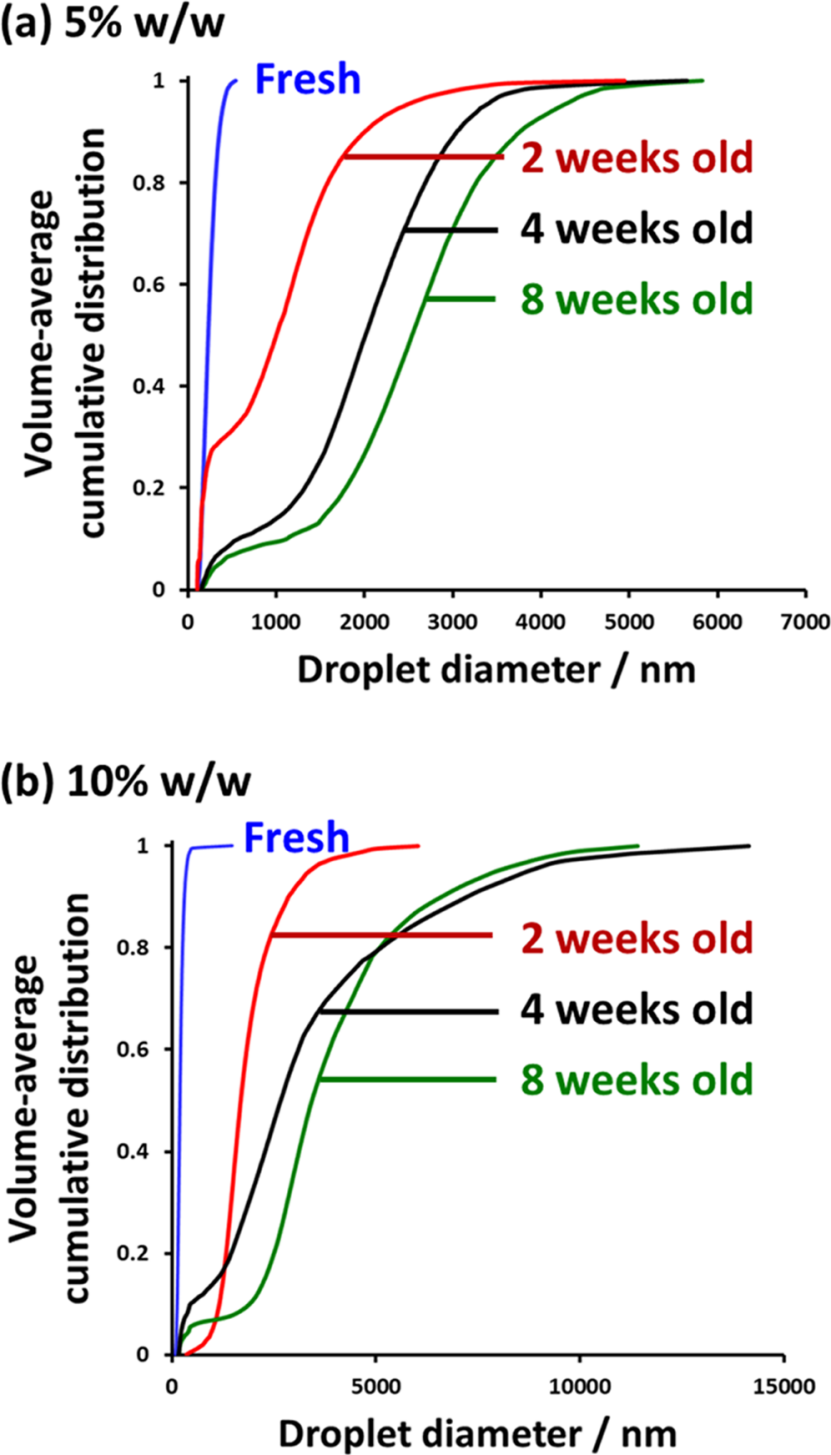
Cumulative droplet size distributions for fresh and aged *n*-dodecane-in-water Pickering nanoemulsions prepared with either (a) 5.0% w/w or (b) 10% w/w core crosslinked PDMAC_77_-PDAAM_40_ diblock copolymer nanoparticles, as determined by analytical centrifugation.

To examine how the copolymer concentration affects the long-term stability of the Pickering nanoemulsions prepared using the core-crosslinked nanoparticles, the change in the mean droplet diameter on aging for up to 8 weeks at 20 °C was monitored using analytical centrifugation. Cumulative droplet size distributions for both fresh and aged nanoemulsions prepared using either 5 or 10% w/w nanoparticles are shown in Figure 8. The droplet size distribution was initially unimodal but gradually became bimodal during aging. Thus, plotting *r*^3^ against time produced a nonlinear relationship in both cases (Figure 6). Nevertheless, the extent of droplet growth could be assessed. Ostwald ripening is clearly more rapid for the nanoemulsion prepared at the higher copolymer concentration, with only approximately 30% of the droplets remaining below 3 μm after 8 weeks. In contrast, more than 70% of the droplets remain below 3 μm for the 5% w/w nanoemulsion over the same aging period. This is perhaps surprising given that such Pickering nanoemulsions are prepared using core-crosslinked nanoparticles. This suggests that excess nanoparticles, like the linear amphiphilic diblock copolymer chains, also promote faster oil transport through the aqueous continuous phase.

**Figure 9:**
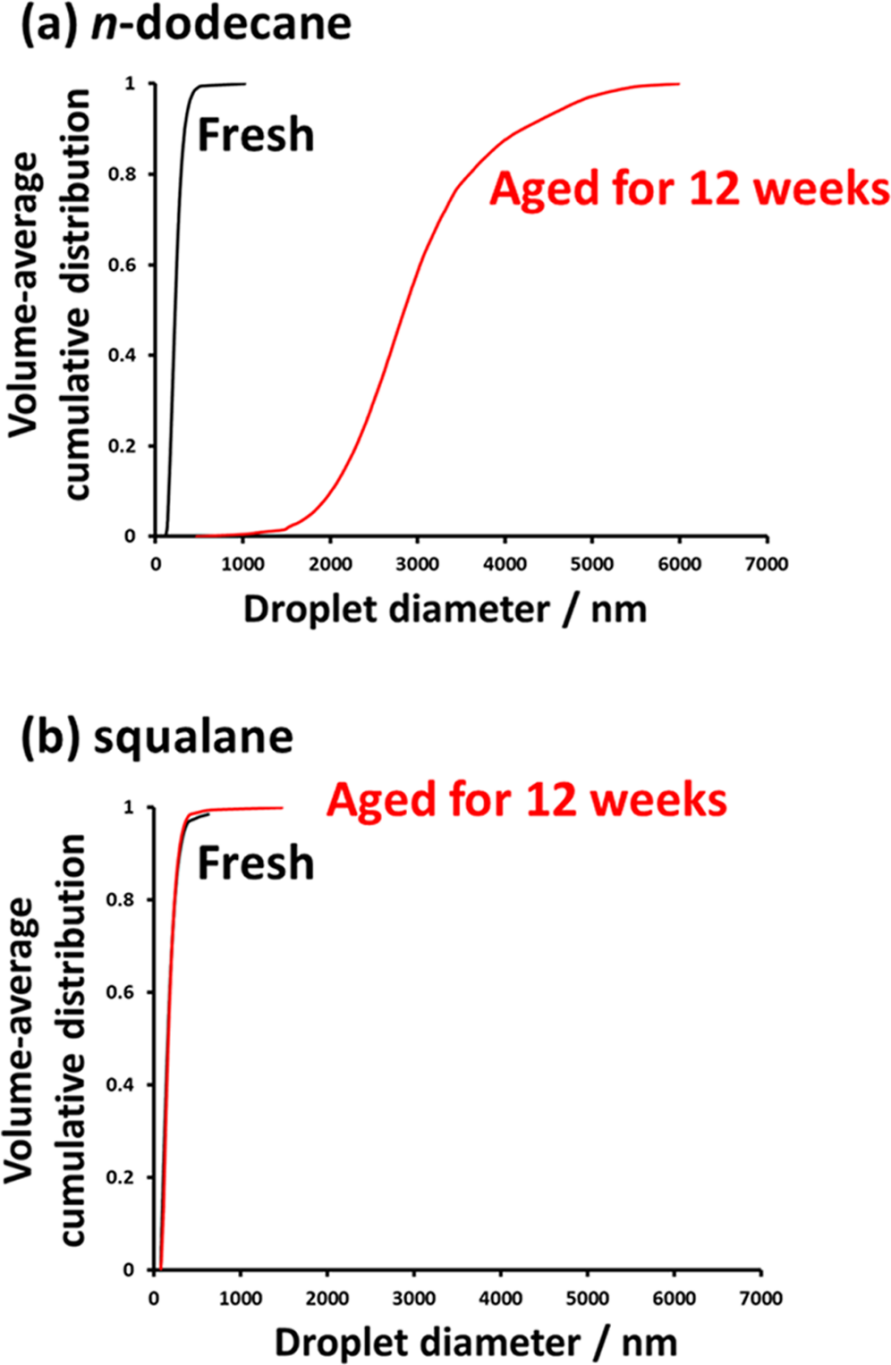
Cumulative droplet size distributions for fresh and 12-week-old oil-in-water nanoemulsions prepared with either (a) *n*-dodecane or (b) squalane using 5.0% w/w linear PDMAC_77_-PDAAM_40_ nanoparticles, as determined by analytical centrifugation.

It is well known that surfactant-stabilized nanoemulsions comprising oils of relatively low aqueous solubility undergo Ostwald ripening on relatively slow time scales.^24,40,46^ This is also the case for Pickering nanoemulsions stabilized using either silica^9^ or diblock copolymer nanoparticles.^22^ More specifically, Persson et al. found that oil-in-water Pickering nanoemulsions prepared with silica nanoparticles yielded highly unstable droplets when using various *n*-alkanes as the oil phase but relatively stable droplets when using squalene, which is a highly water-insoluble naturally occurring oil.^9^ In the current study, Pickering nanoemulsions have been prepared using squalane, which is the hydrogenated derivative of squalene. The aqueous solubility of squalene (0.012 μg dm^–3^)^9^ is significantly lower than that of *n*-dodecane (3.4 μg dm^–3^).^22^ We posit that the aqueous solubility of squalane is comparable to that of squalene. If so, then eq 1 predicts that Ostwald ripening should be substantially suppressed for squalane-based nanoemulsions compared to the corresponding *n*-dodecane-based nanoemulsions. Analytical centrifugation data were obtained for both freshly made and aged nanoemulsions prepared using linear PDMAC_77_-PDAAM_40_ nanoparticles and either *n*-dodecane or squalane as the oil, as shown in Figure 9. In both cases, the droplet size distributions remain almost unchanged after aging for several weeks. Clearly, the rate of Ostwald ripening is significantly lower for nanoemulsions prepared using squalane than those prepared using *n*-dodecane, as demonstrated by the approximately constant volume-average droplet diameter of around 170 nm. This suggests that squalane has a much lower aqueous solubility within the continuous phase than *n*-dodecane.

## Conclusions

A water-soluble PDMAC_77_ precursor was chain-extended via RAFT aqueous dispersion polymerization of DAAM to produce spherical nanoparticles of ∼30 nm diameter. Covalent stabilization of such nanoparticles was achieved at 20 °C using adipic acid dihydrazide. Both linear and core-crosslinked nanoparticles were used in turn to produce *n*-dodecane-in-water nanoemulsions via high-pressure microfluidization processing of precursor macroemulsions prepared using excess nanoparticles. DLS studies confirmed that oil droplets of ∼200–250 nm diameter are produced in both cases. For the core-crosslinked nanoparticles, TEM studies revealed that the original superstructure (i.e., a spherical monolayer of close-packed nanoparticles) was preserved under ultrahigh vacuum conditions, confirming the particle-stabilized (or Pickering) nature of such nanoemulsions. In contrast, TEM studies of nanoemulsions prepared using linear nanoparticles indicated a smooth, featureless structure. Moreover, laser diffraction studies indicated that the droplet size was almost independent of the copolymer concentration. These results indicate that nanoparticle disassembly occurred during microfluidization. Analytical centrifugation was employed to assess the long-term stability of both types of nanoemulsions. An appreciably faster Ostwald ripening was observed for the copolymer chain-stabilized nanoemulsions compared to Pickering nanoemulsions prepared under identical conditions. This is attributed to the more efficient transport of oil through the aqueous phase, which is facilitated by the presence of excess (non-adsorbed) copolymer. Indeed, faster rates of Ostwald ripening were observed when nanoemulsions were prepared using relatively high concentrations of either linear or core-crosslinked nanoparticles. Finally, oil-in-water nanoemulsions were also prepared using squalane, which has a significantly lower aqueous solubility than *n*-dodecane. Squalane-in-water nanoemulsions prepared with either linear or core-crosslinked nanoparticles were much more stable than the corresponding *n*-dodecane-in-water nanoemulsions.

## Supporting Information Available

Full experimental details; ^1^H NMR spectra for the PDMAC_77_ precursor and PDMAC_77_-PDAAM_40_ diblock copolymers; GPC data for the PDMAC_77_ precursor and PDMAC_77_-PDAAM_40_ diblock copolymers; concentration dependence of the droplet diameter of a series of oil-in-water precursor macroemulsions prepared using either the linear or the core-crosslinked nanoparticles as an emulsifier (PDF)
