# Supplementary material for: Long-Term Stability of Pickering Nanoemulsions Prepared Using Diblock Copolymer Nanoparticles: Effect of Nanoparticle Core Crosslinking, Oil Type, and the Role Played by Excess Copolymers
Source: Langmuir. 2022 Jun 23;38(26):8021–9. doi: 10.1021/acs.langmuir.2c00821 (PMC9261185; doi:10.1021/acs.langmuir.2c00821)
Supplement: Supplementary file 1 — la2c00821_si_001.pdf [file la2c00821_si_001.pdf]

Supporting Information for:

Long-term stability of Pickering nanoemulsions prepared using diblock copolymer nanoparticles: effect of nanoparticle core crosslinking, oil type and the role played by excess copolymer

Saul J. Hunter and Steven P. Armes*

[†]Department of Chemistry, Dainton Building, University of Sheffield,
Brook Hill, Sheffield, South Yorkshire, S3 7HF, UK.

Table of Contents

**Experimental Section**		**S2**
**Figure S1.**	^{1}H NMR spectra	**S7**
**Figure S2.**	DMF GPC curves	**S8**
**Figure S3.**	Concentration dependence of droplet diameter for macroemulsions	**S9**

EXPERIMENTAL

Materials. 2-(Dodecylthiocarbonothioylthio)-2-methylpropionic acid (DDMAT), *N,N*-dimethylacrylamide (DMAC), 2,2′-azobis-(2-methylpropionitrile) (AIBN), 1,4-dioxane, *n*-dodecane and squalane were purchased from Sigma-Aldrich (UK) and were used as received. Diacetone acrylamide (DAAM), adipic acid dihydrazide (ADH) and 4,4′-azobis(4-cyanovaleric acid) (ACVA) were purchased from Alfa Aesar (U) and were used as received. Diethyl ether was purchased from Fisher Scientific (UK). All solvents were HPLC-grade and deuterated methanol was purchased from Cambridge Isotope Laboratories (UK). Deionized water was used to prepare all aqueous solutions.

Polymer Characterization

^{1}H NMR Spectroscopy. All NMR spectra were recorded in deuterated methanol (CD_3OD) at 25 °C using a 400 MHz Bruker Avance III HD 400 spectrometer (64 scans were required to ensure high-quality spectra).

Gel Permeation Chromatography (GPC). Copolymer molecular weight distributions were assessed using an instrument comprising two Agilent PL gel 5 μm Mixed-C columns and a guard column connected in series to an Agilent 1260 Infinity GPC system equipped with both refractive index and UV−visible detectors (only the refractive index detector used) operating at 60 °C. The GPC eluent was HPLC-grade DMF containing 10 mM LiBr at a flow rate of 1.0 mL min^{-1}. DMSO was used as a flow-rate marker. Calibration was achieved using a series of near-monodisperse poly(methyl methacrylate) standards (ranging in M_p values from 645 g mol^{-1} to 618 000 g mol^{-1}). Chromatograms were analyzed using Agilent GPC/SEC software.

Dynamic Light Scattering (DLS). A Malvern Zetasizer NanoZS instrument was used to determine the z-average hydrodynamic diameter of diblock copolymer nano-objects at 25 °C

via the Stokes−Einstein equation, which assumes perfectly monodisperse, non-interacting spheres. All measurements were made on either 0.1% w/w copolymer dispersions or 0.1% nanoemulsions in either acidic aqueous solution (pH 2.5) or methanol using disposable plastic cuvettes. Data were averaged over three consecutive runs.

Transmission Electron Microscopy (TEM). Copper/palladium TEM grids (Agar Scientific, UK) were coated in-house to yield a thin film of amorphous carbon. The grids were then subjected to a glow discharge for 30 s. Individual 10 μL droplets of either 0.1% w/w aqueous copolymer dispersions or 0.1% v/v nanoemulsions were placed on freshly-treated grids for 1 min and then carefully blotted with filter paper to remove excess solution. To ensure sufficient electron contrast, a 9.0 μL droplet of a 0.75% w/w uranyl formate was placed on the sample-loaded grid for 20 s and then carefully blotted to remove excess stain. Each grid was then dried using a vacuum hose. Imaging was performed using a FEI Tecnai Spirit 2 microscope fitted with an Orius SC1000B camera operating at 80 kV.

Analytical Centrifugation. Droplet size distributions were assessed using a LUMiSizer analytical photocentrifuge (LUM GmbH, Berlin, Germany) at 20 °C. Measurements were conducted on dilute Pickering nanoemulsions (1.0% v/v *n*-dodecane or squalane) in 2.0 mm path length polyamide cells at 400 rpm for the first 200 profiles (allowing 10 s between each profile), then the rate of centrifugation was increased up to 4000 rpm for a further 800 profiles. The relatively slow initial rate of centrifugation enabled detection of any larger oil droplets that could be present within the nanoemulsion. The LUMiSizer instrument employs space- and time-resolved extinction profiles (STEP) technology to measure the intensity of transmitted near-infrared light as a function of time and position over the entire cell length simultaneously. The gradual progression of these transmission profiles contains information on the rate of creaming of the oil droplets and hence enables assessment of the droplet size distribution. The particle density is an essential input parameter for analytical centrifugation

studies. The droplet density used for the nanoemulsion ageing studies was either the density of *n*-dodecane (0.75 g cm^{-3})[65] or that of squalane (0.81 g cm^{-3}).[66] This assumption ignores any contribution to the droplet density from the adsorbed $PDMAC_{77}$-$PDAAM_{40}$ nanoparticles, but this approximation is reasonable given that we merely wish to assess *relative* changes in the droplet size distribution over time. Undersizing can occur if the droplet concentration is too high owing to the phenomenon of hindered creaming.[27, 75] However, if the droplet concentration is too low, such dilute emulsions scatter light only rather weakly and hence fall outside of the optimum transmission range required for the LUMiSizer instrument (i.e. less than 30% transmission). Given these conflicting requirements, a nanoemulsion concentration of 1.0% v/v was found to be optimal.[25] In the current study, this concentration was used for all analytical centrifugation measurements.

Synthesis of the Poly(*N,N*-dimethylacrylamide) (PDMAC) Precursor via RAFT Solution Polymerization. A typical protocol for the synthesis of a $PDMAC_{77}$ precursor was conducted as follows. 2-(Dodecylthiocarbonothioylthio)-2-methylpropionic acid (DDMAT) (2.10 g, 5.76 mmol), AIBN (95.0 mg 0.576 mmol, CTA/AIBN molar ratio = 10.0), and DMAC (40.0 g, 0.404 mol; target DP = 70) were weighed into a 250 mL round-bottomed flask. 1,4-Dioxane (98.5 mL) was added to produce a 30% w/w solution, which was purged with nitrogen for 30 min. The sealed flask was immersed into an oil bath set at 70 °C for 80 min (final DMAC conversion = 93%, as judged by ^{1}H NMR spectroscopy), and the polymerization was subsequently quenched by immersing the flask in ice, followed by exposure to air. 1,4-Dioxane (100 mL) was added to the reaction solution, followed by precipitation into a ten-fold excess of diethyl ether. The precipitate was redissolved in 1,4-dioxane and precipitated once more into excess diethyl ether. The crude precursor was then dissolved in deionized water, any residual diethyl ether/dioxane was removed under reduced pressure, and the resulting aqueous solution was freeze-dried for 48 h. The purified PDMAC precursor was obtained as a yellow solid. End-

group analysis using UV spectroscopy indicated a mean degree of polymerization of 77. DMF GPC analysis indicated an M_n of 7 300 g mol^{-1} and an M_w/M_n of 1.19, respectively.

Synthesis of Linear PDMAC$_{77}$–PDAAM$_{40}$ Diblock Copolymer Spherical Nanoparticles by RAFT Aqueous Dispersion Polymerization of DAAM at pH 2.5. The typical protocol for the synthesis of PDMAC$_{77}$–PDAAM$_{40}$ spherical nanoparticles at 10% w/w solids was as follows. PDMAC$_{77}$ precursor (0.998 g, 0.125 mmol), ACVA (3.5 mg, 12.5 μmol, CTA/ACVA molar ratio = 10), and DAAM monomer (0.844 g, 5.0 mmol; target DP = 40) were weighed into a 50 mL round bottom flask. Deionized water (16.58 g) was then added to afford a 10% w/w aqueous solution, which was adjusted to pH 2.5 using HCl and then degassed for 30 min prior to immersion in an oil bath set at 70 °C. This reaction mixture was stirred for 4 h and then the polymerization was quenched by exposure of the flask contents to air while cooling to 20 °C. The DAAM conversion was greater than 99% as judged by ^{1}H NMR spectroscopy. DMF GPC analysis indicated an M_n of 12 500 g mol^{-1} and an M_w/M_n of 1.30, respectively.

Nanoparticle Core Crosslinking Using ADH. The protocol used for core-crosslinking the PDMAC$_{77}$–PDAAM$_{40}$ spheres was as follows. A 20% w/w aqueous dispersion of PDMAC$_{77}$–PDAAM$_{40}$ spheres (2.50 g; prepared according to the above protocol) and adipic acid dihydrazide (ADH; 0.012 g, 0.068 mmol, DAAM/ADH molar ratio = 10.0) were added to a 14 mL glass vial. This reaction mixture was then stirred at 25 °C for 6 h, which is typically sufficient time for ADH crosslinking of the nanoparticle cores to occur.[64]

Preparation of PDMAC$_{77}$–PDAAM$_{40}$-Stabilized Macroemulsions Using High-Shear Homogenization. A 5.0% w/w aqueous dispersion of either linear or ADH-crosslinked PDMAC$_{77}$–PDAAM$_{40}$ spheres (4.0 mL, pH 2.5) was added to a 14 mL glass vial and homogenized with *n*-dodecane (1.0 mL) at 13 500 rpm for 2.0 min using an IKA Ultra-Turrax T-18 homogenizer equipped with a 10 mm dispersing tool and operating at 20 °C.

Preparation of $PDMAC_{77}$–$PDAAM_{40}$-Stabilized Nanoemulsions by High-Pressure Microfluidization. A Pickering macroemulsion (5.0 mL, initial nanoparticle concentration in the aqueous phase = 5.0% w/w) prepared using either linear or ADH-crosslinked $PDMAC_{77}$–$PDAAM_{40}$ spherical nanoparticles was further processed using an LV1 low-volume microfluidizer processor (Microfluidics, USA). The applied pressure was fixed at 30 000 psi and each coarse precursor emulsion was passed eight times through the LV1 unit to achieve well-defined nanoemulsions.

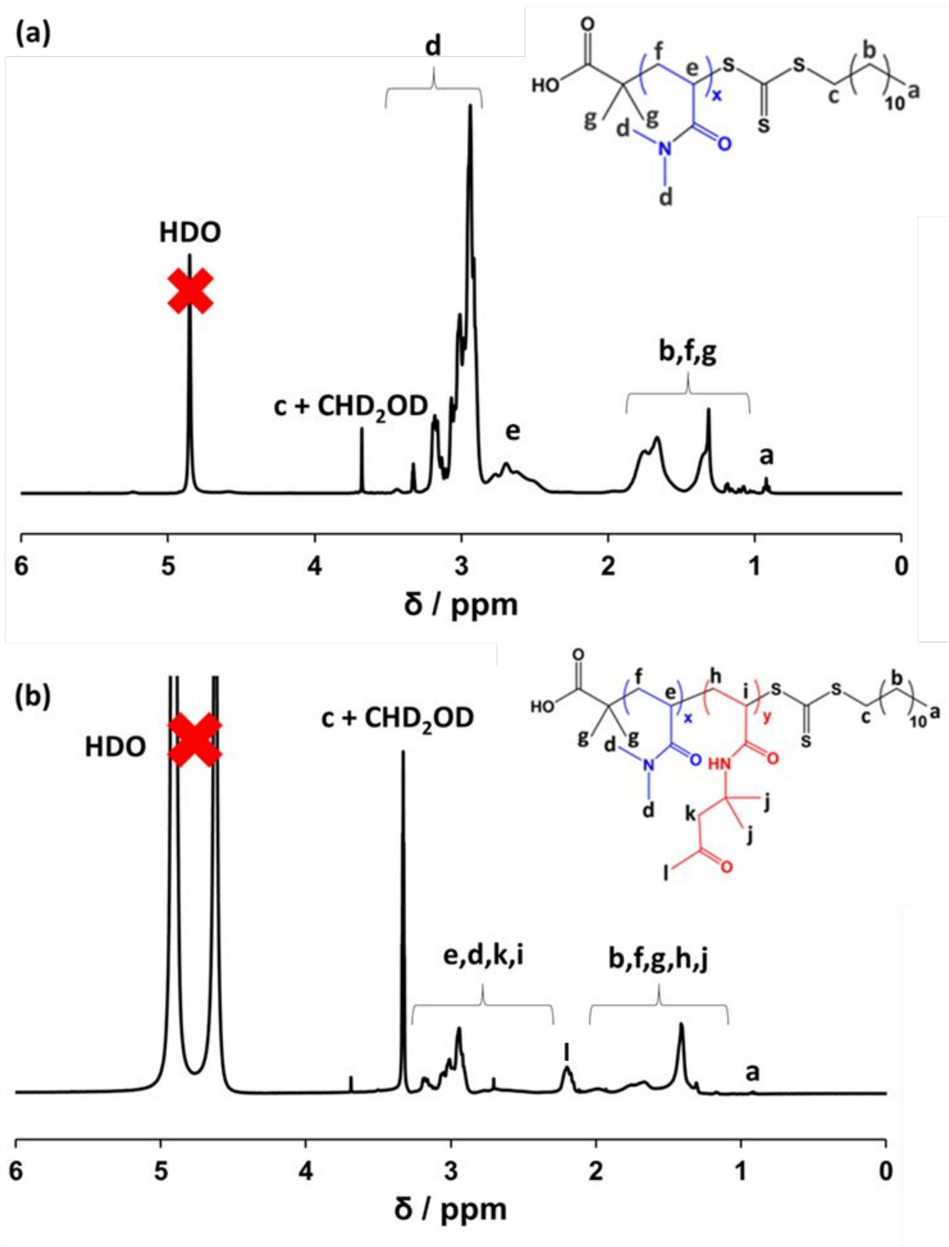

Figure S1. ^{1}H NMR spectra (CD_3OD) recorded for (a) a $PDMAC_{40}$ precursor and (b) a $PDMAC_{77}$-$PDAAM_{40}$ diblock copolymer.

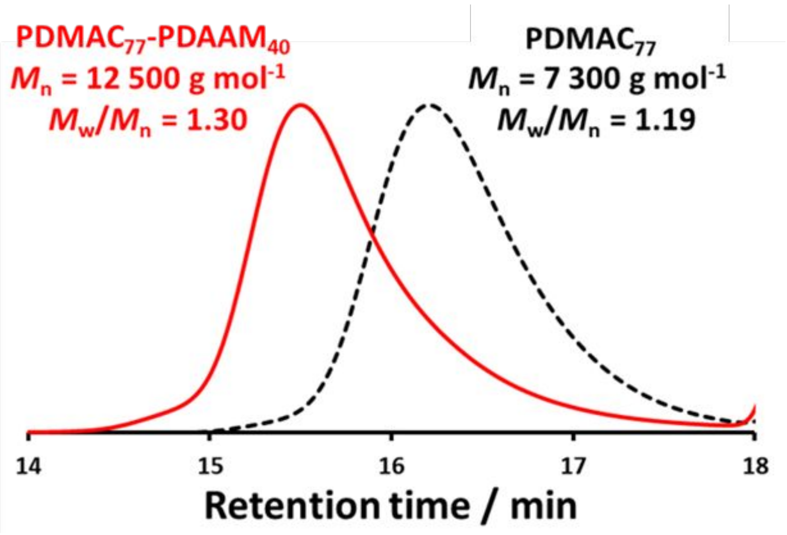

Figure S2. DMF GPC curves (refractive index detector) recorded for the $PDMAC_{77}$ precursor (black trace) and the corresponding $PDMAC_{77}$–$PDAAM_{40}$ diblock copolymer (red trace) prepared via RAFT solution polymerization and RAFT aqueous dispersion polymerization, respectively.

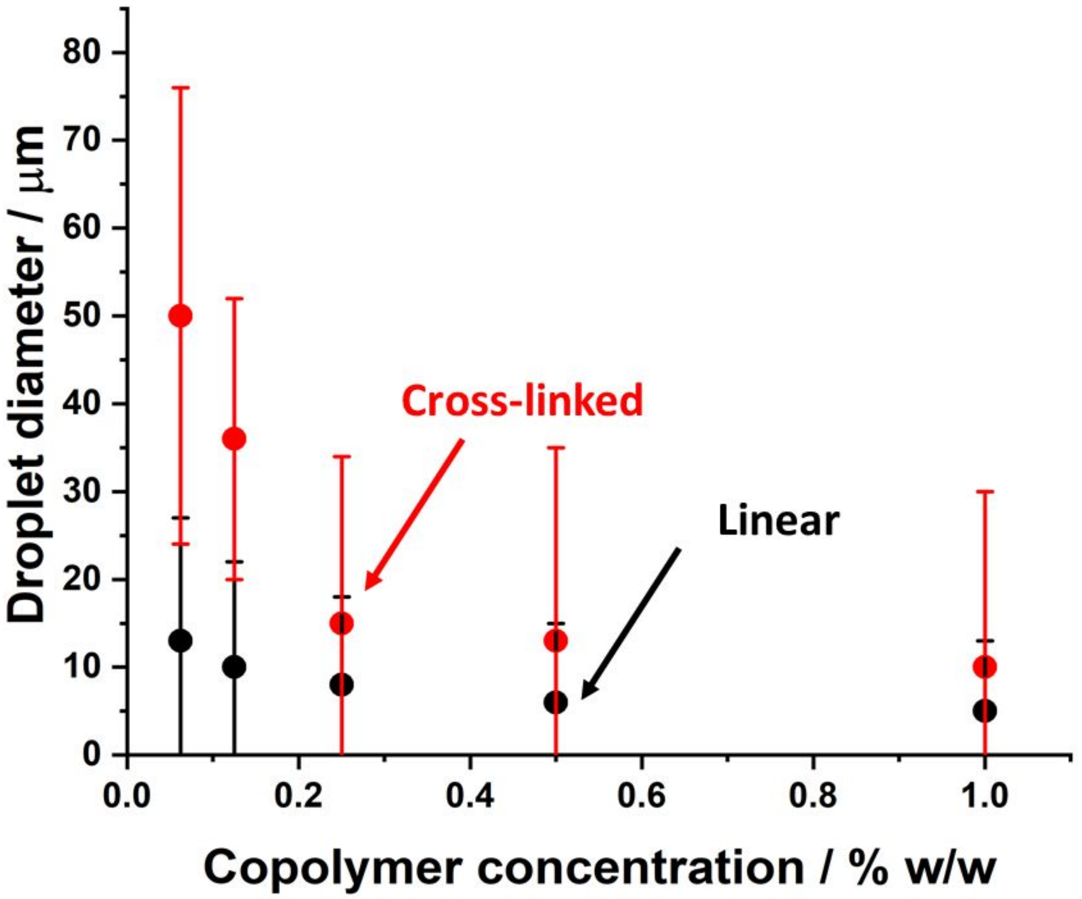

Figure S3. Concentration dependence of the droplet diameter determined by laser diffraction studies of a series of *n*-dodecane-in-oil precursor Pickering macroemulsions prepared via high-shear homogenization of *n*-dodecane (volume fraction = 0.20) at 13 500 rpm for 2 min at 20 ^{o}C when using either the linear or the core-crosslinked nanoparticles as an emulsifier.
